# The Impact of Academic Procrastination on Second Language Writing: The Mediating Role of L2 Writing Anxiety

**DOI:** 10.3389/fpsyg.2022.851120

**Published:** 2022-05-04

**Authors:** Chen Zhang, Wenzhong Zhang

**Affiliations:** College of Foreign Languages, Nankai University, Tianjin, China

**Keywords:** academic procrastination, L2 writing, anxiety, readability, mediating role

## Abstract

Recently, there has been a surge of interest in the exploration of psychological properties in a second language context. Considerable literature has grown up around the influence of these psychological properties on L2 writing specifically. However, the impact of academic procrastination, which is an important psychological property, has been understudied and it remains unclear how affective factors in L2 might play a role in the above potential influence on L2 writing. Therefore, the current study explored the impact of academic procrastination on L2 writing and examined the mediating role of L2 writing anxiety, by adopting text readability as an innovative approach to assessing L2 writing performance. Participants were 55 Chinese speakers of L2 English. By utilizing the collected questionnaire data and the readability indicators of the L2 writing task, the current research conducted correlation analysis, regression analysis, and structural equation modeling analysis. The results revealed that academic procrastination had a significant negative impact on the readability indicator of Flesch-Kincaid Grade Level in L2 writing. L2 writing anxiety played a complete mediating role in the impact. Academic procrastination can significantly affect Flesch-Kincaid Grade Level of L2 writing indirectly through L2 writing anxiety. Pedagogical implications and future studies were discussed.

## Introduction

Recent years have witnessed a growing interest in the roles that affective factors play in second language learning. Among these domain-specific affective factors, L2 learning motivation, L2 learning anxiety, L2 learning self-efficacy, and willingness to communicate in L2 are the foci of research and have been extensively discussed (Woodrow, 2011; Yim, 2014; Joe et al., 2017; Zhang et al., 2020; Turner et al., 2021; Wang et al., 2021). With the extension of research horizon and investigation, domain-general psychological properties and learner’s personality traits are gradually integrated into L2 research, and their impacts on L2 learning have attracted much attention (Jiang and Dewaele, 2019; Lan et al., 2021; Liu et al., 2021). Previous research has probed into the properties such as boredom (Li, 2021), resilience (Danesh and Shahnazari, 2020), emotional intelligence (Ożańska-Ponikwia, 2018) and enjoyment (Boudreau et al., 2018) etc. Nonetheless, academic procrastination, as an important psychological property, is understudied and has not been empirically and systematically investigated in the L2 learning context. Existing evidence revealed that academic procrastination is extremely prevalent in higher education. Steel (2007) reported that 80%–95% of college students had experienced procrastination and 50% of them believed that procrastination in coursework had perplexed them consistently. Given this ubiquity, it is highly likely that academic procrastination is influencing a vast majority of L2 learners. However, to date, little evidence has been found associating academic procrastination with L2 learning outcomes and affective constructs.

The current study, therefore, takes the initiative of shifting the focus to the exploration of domain-general psychological property in L2 learning context and demonstrates an interdisciplinary nature. By highlighting the role of academic procrastination within the scope of L2 writing, this study aims to explore the impact of academic procrastination on L2 writing performance, and advance the understanding of its power in the L2 context. Meanwhile, the current research also examines how anxiety mediates the relationship between academic procrastination and L2 writing performance, attempting to generate fresh insights and provide suggestions for L2 writing research and teaching.

## Literature Review

### Psychological Interpretations of Academic Procrastination and Anxiety

Procrastination is the action of unnecessarily postponing the tasks, resulting in discomforts in subjective experience (q.v. Solomon and Rothblum, 1984; Li et al., 2019). It has the characteristics of being voluntary, evasive and irrational (Zhang and Chen, 2013). Academic procrastination is a concrete manifestation of procrastination in academic learning activities and refers to the action of delaying learning-related tasks (McCloskey, 2012), which is particularly prominent in the execution and output stage. Academic procrastination is one of the key concepts in psychological research. Investigations have been conducted to elucidate the basic concept of academic procrastination and explore the relationships between academic procrastination and academic achievement, time management, perfectionism and parenting styles. Previous research revealed that academic procrastination and academic achievement were negatively correlated (Kim and Seo, 2015; Asio, 2020). Meanwhile, a negative correlation was also found between academic procrastination and time supervision (Zhou et al., 2014; Li et al., 2018). Academic procrastinators are usually perfectionists (Chen et al., 2013; Liu et al., 2020). Encouraging response, attention and tolerance provided by parents could contrarily predict students’ level of academic procrastination on a significant level (Zakeri et al., 2013; Zhang and Chen, 2013).

Apart from the aforementioned factors, academic procrastination is also closely associated with anxiety. As a psychological term, anxiety refers to an unpleasant feeling which is accompanied by tension and characterized by inner turmoil, apprehension and fear (Oltmanns and Emery, 2019). Anxiety can trigger negative behaviors such as rumination and hyper-stress response, and somatic symptoms such as insomnia and tachycardia. According to the Broaden-and-Build Theory (Fredrickson, 2001), anxiety, as a typical negative emotion, would constrict the resources of cognition and impose restrictions on the advancement of learners’ ability to focus on new things, which is not conducive to long-term resource construction.

### The Interdependency Between Academic Procrastination and Anxiety: A Theoretical Account

Academic procrastination and anxiety are closely correlated, in the sense that they share expectancy and value appraisals as antecedents. In terms of procrastination behaviors, especially in academic settings, expectancy and value appraisals are the predominant factors. Theoretical enquiries into academic procrastination have a long history. Investigations include the psychoanalytic and psychodynamic approach, the behavioristic approach, the cognitive approach and the temporal motivation theory, among which the temporal motivation theory is the most comprehensive meta-framework to expound on the nature of procrastination to date (Siaputra, 2010). Temporal motivation theory posits that four determinants are particularly influential in predicting the utility in accomplishing tasks. They are expectancy, value, impulsiveness and delay, respectively. Expectancy refers to the odds and probability that one could successfully achieve, while value indicates the subjective idea or preference on the benefit or pleasantness of a certain activity. Impulsiveness implies the sensitivity to delay (i.e., the propensity to short-term gain) whereas delay denotes the actual duration to attain the final expected reward. As illustrated in the analytical equation below, expectancy and value act as numerators, and impulsiveness and delay serve as the denominators. Higher expectancy and value, and lower impulsiveness and delay could contribute to a stronger motivation or a greater utility of fulfilling tasks.


$$\begin{array}{c} Motivation\;(Utility)=\frac{Expectancy\times Value}{1+Impulsiveness\times Delay}\\ (\text{Steel et al., }2018,\text{ p. }2). \end{array}$$

The temporal motivation theory claims that motivation and utility have a close correlation with setting priorities of task completion. People incline to procrastinate when low utility is perceived (Siaputra, 2010). Since expectancy and value appraisals are the prime determinants of assessing the motivation and utility of task accomplishment, they are the antecedents of procrastination behaviors as well. High expectancy and value appraisal would keep procrastination at a lower level, and vice versa.

As regards anxiety, expectancy and value appraisals are also within the central discussion, guided by the control-value theory (Pekrun, 2006). The control-value theory is a crucial contemporary theory in the field of educational psychology and seeks to illuminate achievement emotions in academic settings (Putwain et al., 2018; Li, 2021). Anxiety is a frequently experienced and typical kind of achievement emotion which is generated from achievement activities and outcomes (Pekrun and Perry, 2014). The control-value theory posits that control and value appraisals are strongly associated with achievement emotions. Subjective control refers to “the perceived causal influence of an agent over actions and outcomes” (Pekrun, 2006, p. 317), in which expectancies are the central issue to address. Subjective value indicates “the perceived valence of actions and outcomes” and “the perceived importance of success” (Pekrun, 2006, p. 317). Intrinsic and extrinsic value appraisals are proposed as two main categories. From the perspective of control-value theory, anxiety could be triggered if negative valence of outcomes is perceived and, in the meantime, the controllability is at a medium level. Specifically, if one has a lower situation-outcome expectancy [i.e., “appraisals of external control over outcomes” (Pekrun, 2006, p. 318)], one’s intrinsic and extrinsic appraisals of values toward the task might be underestimated, and one’s action-control (e.g., self-efficacy) and action-outcome expectancies (i.e., the produced outcomes which based on concrete actions) might, in turn, be lowered down. If one holds to be uncertain in achieving positive outcomes or realizing preventive actions, anxiety is instigated accordingly.

In a more profound sense, academic procrastination and anxiety coexist and are interdependent. The “Appraisal-Anxiety-Avoidance” (AAA) model of procrastination (Milgram and Toubiana, 1999) provided insights into the role of anxiety in procrastination behavior. When individuals feel incompetent or threatened after the appraisal of the pending tasks in academic settings, they would feel anxious and therefore avoid completing the tasks, so as to mitigate the apprehensive mood, which leads to the occurrence of procrastination. The interpretation of impulsiveness in the temporal motivation theory also sheds light on the close connection between anxiety and procrastination. Impulsiveness decides our response to anxiety in general (Steel, 2011, p. 13). For people whose impulsiveness is maintained at a higher level (i.e., whose gratification is less likely to be delayed), anxiety over a deadline will immediately provoke procrastination (Steel, 2011, p. 13).

Procrastination is usually utilized as a mechanism to resist anxiety. Even though anxiety can be temporarily alleviated by procrastination, it would soon be raised to a higher level, since the pending state of the academic tasks would arouse even more severe anxiety. Previous research demonstrated the close connection between procrastination and anxiety, depression and lower self-esteem (Onwuegbuzie, 2004; Cavusoglu and Karatas, 2015). Once students wish to mitigate the anxiety caused by fear of difficulties and procrastinate, anxiety would not disappear, but become more serious. The vicious circle is thus formed.

### Academic Procrastination and Anxiety in L2 Writing

In L2 context, especially in L2 writing, academic procrastination and anxiety both play critical roles. Previous research has shown that academic procrastination in L2 learning can be influenced by internal factors, such as fear of failure, self-efficacy and self-imposed limitations, as well as external factors, such as the role of teacher, task aversiveness and the timing for rewards and punishment (Martínez and Witten, 2013). In the meantime, factors such as time management, sincerity, personal initiative can also influence the level of academic procrastination in foreign language learning (Wirajaya et al., 2020). It is worth noting that academic procrastination can negatively predict foreign language learning on a significant level (Zhong, 2017; Aydoğan and Akbarov, 2018; Yurtseven and Akpur, 2018; Qi, 2020). Additionally, Fritzsche et al. (2003) analyzed the relationship between academic procrastination and writing and found that procrastination in writing tasks was closely connected with general anxiety, writing anxiety, writing dissatisfaction and low scores in writing. Although the above study didn’t probe into the L2 writing context, it still provided support and evidence and contributed to framework establishment. Kafipour and Jafari (2021) introduced academic procrastination into L2 writing research and suggested that there was a significant negative correlation between academic procrastination and L2 writing. In other words, the higher the level of academic procrastination, the lower the level of L2 writing. This study explained the influence of academic procrastination in L2 writing, however, the research methodology and data analysis were relatively simple, and the examination of L2 writing only relied on overall scores. Given that research on academic procrastination in L2 writing context was understudied and the indicators adopted were quite limited, it is critically important to further explore the impact of academic procrastination on L2 writing, to generalize abundant analyses and enrich the discussion.

Anxiety is frequently highlighted in the language learning environment as well (Horwitz et al., 1986; Gardner and MacIntyre, 1993; Hilleson, 1996) and is embodied in L2 writing as a continuous mental state of unreadiness or not feeling well written (Daly and Miller, 1975). It is also manifested in the sense that the fear of writing exceeded the expectation of improving writing skills (Thompson, 1980). Anxiety is closely associated with L2 learning and usually in a negative way (Zabihi et al., 2021). A higher level of anxiety in language learning could exert a significant negative impact on language input, processing and output (MacIntyre and Gardner, 1994). In L2 writing context, the role of anxiety is highly prominent and can potentially be probed from several sub-dimensions. In the early exploration of anxiety in L2 writing, Daly and Miller (1975) made an initial attempt by devising the measuring instrument of *Writing Apprehension Test* (WAT). Although WAT contributed to a deep understanding of anxiety in L2 writing, it suffered from flaws in construct validity. According to the analyses from following empirical studies (Burgoon and Hale, 1983; Cheng et al., 1999), WAT had a multi-dimensional nature and encompassed several other properties, such as self-efficacy and self-confidence beliefs, enjoyment, discomfort, etc., apart from anxiety (Cheng, 2004). Thus, it was difficult to disentangle the sub-dimensions of anxiety in L2 writing by referring to WAT. For the sake of unfolding the attributes of L2 writing anxiety, Cheng (2004) conducted a factor analysis to conceptualize L2 writing anxiety and proposed a three-dimensional taxonomy, which is widely adopted within the field at present. In the conceptualization, somatic anxiety, avoidance behavior and cognitive anxiety are the general components. Somatic anxiety referred to the unpleasant somatic experience aroused by the autonomic nervous system, such as stomach discomfort, tachycardia, excessive sweating, nervousness, and numbness, etc. Avoidance behavior encompassed actions and behaviors such as withdrawal, avoidance and procrastination. Cognitive anxiety referred to the psychological experience of anxiety, including the obsession with performance, negative expectations, and attention to other people’s opinions (Cheng, 2004). These three sub-dimensions exhibit independent and distinctive features and establish a full picture for a thorough understanding of L2 writing anxiety.

Past studies have empirically demonstrated and reached a consensus on the negative correlation between L2 writing anxiety and L2 writing performance (Guo and Qin, 2010; Asmari, 2013; Li et al., 2013; Sarkhoush, 2013; Guo, 2018; Zabihi, 2018; Sabti et al., 2019; Saedpanah and Mahmoodi, 2020; Soleimani et al., 2020). However, apart from Zabihi’s (2018) study which examined the complexity, accuracy and fluency features of L2 texts, most research focused on the observation of overall L2 writing performance. Hence, there is still a paucity of studies investigating the impact of L2 writing anxiety on L2 writing performance by multi-dimensioned evaluations, including both external evaluations and inherent features of L2 texts. Meanwhile, no prior studies have particularly addressed the sub-dimensions of anxiety in L2 writing. Thus, more in-depth and detailed exploration is critically required.

Considering the interdependent relationship between academic procrastination and anxiety, and the negative influence of anxiety on L2 production (MacIntyre and Gardner, 1994), it is highly possible that academic procrastination could exert an impact on L2 writing indirectly via anxiety. However, the existing accounts did not provide relevant empirical evidence.

### Readability: An Innovative Approach to Assessing L2 Writing Performance

To assess the writing performance in the second language context, external evaluation is the most frequently utilized measure. In the evaluation, native speakers are usually invited to be the external markers and assess the works of writing from L2 learners according to the given rubrics. The applied criteria include task achievement, coherence and cohesion, lexical resources and grammatical range and accuracy, etc. An overall score or the score from each benchmark is used to represent the learner’s performance in L2 writing. Albeit the validity and efficiency, external evaluations suffered from markers’ subjective judgments and preferences to some extent. Hence, an approach of assessment that could provide objective observations is urgently required. Internal evaluation, which could probe into the inherent L2 text features, is considered to be an alternative to the traditional external evaluation. Due to its objective nature, it has obtained much attention and been applied to a multitude of empirical research. Features such as syntactic and lexical complexities are explored and thus the L2 writing proficiency can be fully revealed from an objective perspective. Commonly adopted syntactic indices involve mean length of sentence, clause per sentence, verb phrase per T-unit, etc. and lexical indices include lexical sophistication, Type/Token ratio (TTR), lexical density and verb diversity, etc.

Apart from these indices, text readability has the potential to represent L2 learner’s proficiency in writing as well. It refers to the level of difficulty when readers read a certain text (Fry, 2002). Originally, text readability was mostly adopted for screening and selecting appropriate materials for L2 learning and testing. However, since its indicators can reflect text features and represent the level of difficulty in vocabulary and sentence usage (Zhang and Li, 2019), text readability can be regarded and utilized as an innovative approach to assessing L2 writing performance. Unfortunately, as an important tool for understanding the inherent features of L2 texts, it has not been included in the evaluation system. And given the current research, it has not been applied in evaluating L2 learners’ writing performance from the perspective of text difficulty in most cases. Therefore, it is worth exploring the writing performance in the L2 context by adopting text readability as the evaluating approach.

Widely accepted methods of assessing text readability include metrics and formulas examining the traditional features, which comprise the number of words per sentence and the number of characters or syllables per word. Flesch Reading Ease and Flesch-Kincaid Grade Level are the tools which explore the above traditional features and enjoyed an enormous popularity in the field. Another approach to the investigation of text readability is machine learning analysis (Xia et al., 2016; Lee and Lee, 2020), which represents the new progress of measurement. This approach demonstrates an improvement of measuring accuracy and covers a wider range of text features, such as language modeling features and discourse-based features, etc. Considering that the current study specifically focused on the examination of the traditional features, Flesch Reading Ease and Flesch-Kincaid Grade Level were utilized. These formulas can be used to reflect text difficulty and readability. The range of Flesch Reading Ease is 0-100. The higher the score of Flesch Reading Ease, the easier the text. In contrast, the range of Flesch-Kincaid Grade Level is 0–12. The lower the score, the easier the text.

The formulas of Flesch Reading Ease and Flesch-Kincaid Grade Level are presented as follows:


Flesch⁢Reading⁢Ease

=206.835-(1.015×ASL)-(84.6×ASW)

Flesch-Kincaid⁢Grade⁢Level

=(0.39×ASL)+(11.8×ASW)-15.59

where ASL represents the average sentence length, and ASW denotes the average number of syllables per word.

In consideration of the literature reviewed, the current study innovatively integrates academic procrastination and L2 writing anxiety in the L2 context, and analyses from a holistic perspective. By constructing the structural equation model, this study adopts text readability indicators to evaluate L2 learners’ writing performance and further explore the impact of academic procrastination on L2 writing and the mediating effect of the multi-dimensioned L2 writing anxiety.

## Research Questions

This study adopted the cross-sectional quantitative research method to explore the impact of academic procrastination on L2 writing performance and examine the mediating role of L2 writing anxiety. Text readability indicators were utilized to examine learners’ performance in L2 writing. English was the target language in the current research.

Specific research questions were:

(1) Can academic procrastination have an impact on L2 writing performance? What is the effect size if the impact exists?
(2) Does L2 writing anxiety play a mediating role in the impact of academic procrastination on L2 writing performance?

## Methodology

### Participants

By adopting the convenient sampling method, the current study recruited 55 Chinese speakers of L2 English as participants. They came from a renowned national key university in China and were second-year undergraduates who specialized in English language and literature at the time of the experiment. All the participants had attended an introductory course of English writing (Phase One) and received systematic training for one semester before they were enrolled in the current study. The instructions involved understanding the thinking pattern of English writing and writing procedures, addressing the audience, punctuations and format mechanics, vocabularies and dictions, modes of development, different genres of English writing and practical writings, etc. During the research, the participants were undertaking the intermediate course of English writing (Phase Two) which focused on critical and argumentative writing.

Scores from the course assessment of English writing (Phase One) revealed that during the early stage of acquiring the writing skills in L2 English, the participants demonstrated proper English writing proficiencies (Full mark: 100; Maximum = 93, Mean = 86.64, SD = 4.102, Median = 87, First quartile = 84). They had an excellent performance in the first phase of English writing study and exhibited great potential in enhancing their writing abilities in L2 English.

### Instruments

Four instruments were selected to contribute to the empirical research. They were the Academic Procrastination Scale, Second Language Writing Anxiety Inventory, Coh-Metrix and the L2 writing task.

#### Academic Procrastination Scale

The Academic Procrastination Scale (APS) utilized in the current research was compiled by McCloskey (2012). 25 items were involved in the scale and covered six aspects which were psychological beliefs about ability, distraction, social factors, time management, personal initiative and laziness, respectively. A 5-point Likert scale (totally disagree, disagree, neutral, agree, totally agree) was treated as the marking reference. APS had quite high reliability (Cronbach’s coefficient α = 0.94).

#### Second Language Writing Anxiety Inventory

Second Language Writing Anxiety Inventory (SLWAI) was established by Cheng (2004). It was a 5-point Likert scale assessing three dimensions which were somatic anxiety, avoidance behavior and cognitive anxiety, respectively. 22 items were involved in the inventory, in which 7 items were for assessing somatic anxiety, 7 items for evaluating avoidance behavior and 8 items for determining cognitive anxiety. The overall reliability of the inventory was quite high (Cronbach’s coefficient α = 0.91), and the reliabilities for each dimension were all above 0.80.

#### Coh-Metrix

Coh-Metrix is an online analyzer designed and developed by McNamara et al. (2014) and is applicable in evaluating the level of coherence and cohesion of written and oral texts. This analyzer can provide researchers and practitioners with real-time analysis in terms of evaluating text difficulty and readability. Coh-Metrix can provide 11 categories of indices, including descriptive data, text easability principal component scores, referential cohesion, latent semantic analysis, lexical diversity, connectives, situation model, syntactic complexity, syntactic pattern density, word information and readability. Within the assessment of text readability, the Flesch Reading Ease and Flesch-Kincaid Grade Level were involved.

#### L2 Writing Task

Students were required to complete an L2 writing task in the current research. The topic was “My favorite restaurant”. The instruction was given as to observe their favorite restaurant and write a short essay of no less than 200 words. Students should fulfill the writing purpose and clarify the reason for choosing the restaurant as their favorite and highlight the detailed description of space.

### Research Steps

Data collection of L2 writing texts and questionnaires, and the evaluation of the collected texts were the important steps in the current study. When collecting L2 writing texts, the researcher assigned the writing task to the participants and provided detailed instructions. After submission, the texts were further processed. After the L2 writing data were collected, participants completed the questionnaire surveys. In the writing text evaluation stage, the researcher utilized Coh-Metrix to analyze the readability of L2 writing texts. All the collected data was later integrated into statistical analysis.

## Results

### The Total Effect of Academic Procrastination on L2 Writing Readability

**FIGURE 1:**
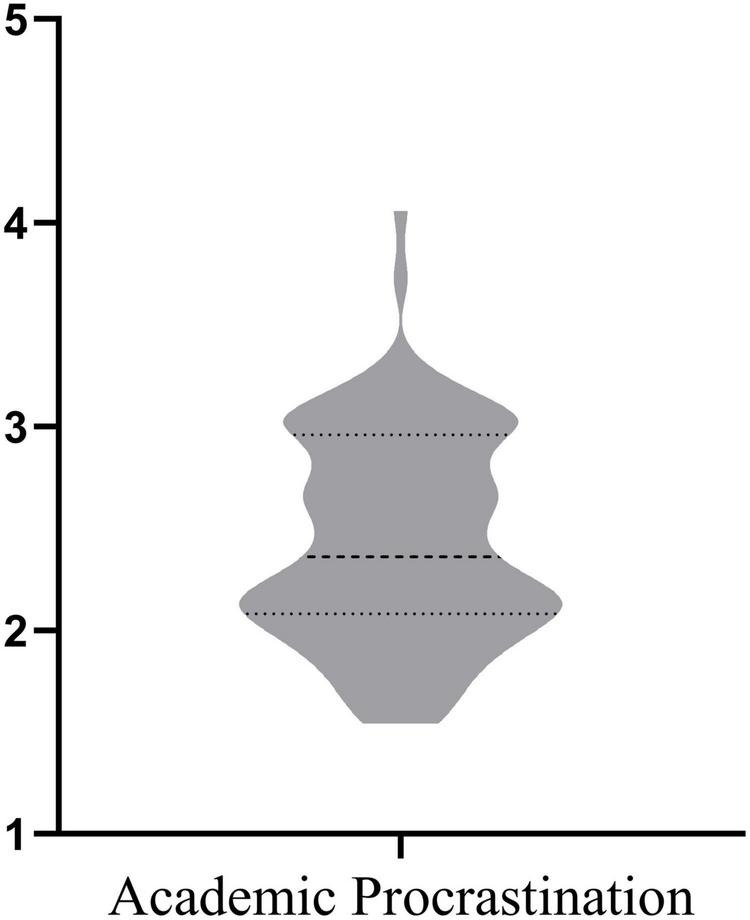
Overview of academic procrastination.

**FIGURE 2:**
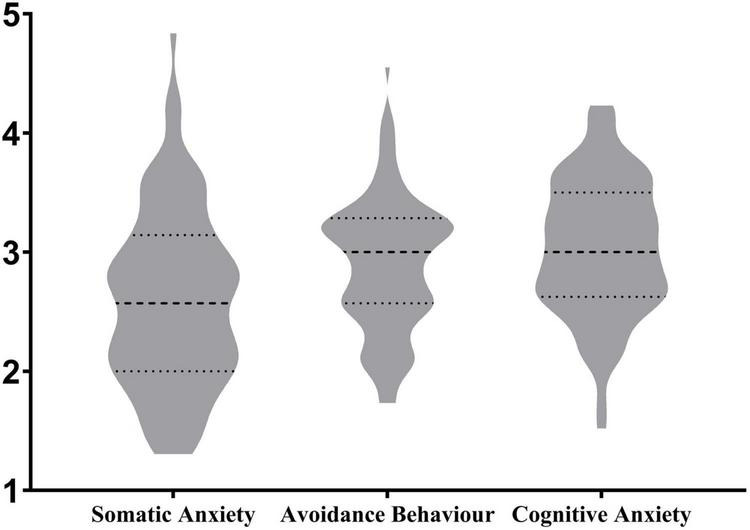
Overview of L2 writing anxiety.

To obtain the general picture of academic procrastination, and L2 writing anxiety from different dimensions, descriptive analyses were conducted by utilizing *SPSS 26* and *GraphPad Prism 9*. The following violin charts (Figures 1, 2) represent the median and interquartile range, and meanwhile show the distribution of the data.

Figure 1 is an overall representation of academic procrastination. According to the scale definition of APS, the higher the rating, the higher level of academic procrastination. Descriptive analyses revealed that participants’ academic procrastination remained at a moderate level in the current study (Mean = 2.454, SD = 0.5539).

Figure 2 displays an overview and the distribution of L2 writing anxieties, including somatic anxiety, avoidance behavior and cognitive anxiety. The chart shows that among the three dimensions of L2 writing anxiety, cognitive anxiety remained at a relatively high level (Mean = 3.027, SD = 0.6013), and was followed by avoidance behavior (Mean = 2.906, SD = 0.6044). The level of somatic anxiety was relatively low (Mean = 2.621, SD = 0.7806). By referring to the scale definition in SLWAI, participants had moderate L2 writing anxiety in general.

In terms of the readability of L2 writing texts, descriptive analyses manifested that the Flesch Reading Ease was at an intermediate level (Mean = 65.276, SD = 7.3382), while the Flesch-Kincaid Grade Level was at the upper-middle level (Mean = 8.236, SD = 1.413). According to the formulas, the lexical and sentence lengths in participants’ texts were relatively short.

To investigate the total impact of academic procrastination on L2 writing readability, correlation analysis and regression analysis were conducted. The results indicated that academic procrastination was not correlated with Flesch Reading Ease (*p* = 0.08 > 0.05) but was negatively correlated with Flesch-Kincaid Grade Level on a significant level (*p* = 0.048 < 0.05). The total effect was −0.268. The difference of the statistical significance of the above correlation analyses might lie in the different weighing factors and coefficients presented in the formulas of Flesch Reading Ease and Flesch-Kincaid Grade Level. To further explore the dependent relationships between academic procrastination and Flesch-Kincaid Grade Level, regression analysis was implemented and showed that academic procrastination can negatively influence Flesch-Kincaid Grade Level on a significant level (*F* = 4.096, *p* = 0.048 < 0.05).

### Analysis of the Mediating Effect

The mediating effect in the impact was examined by utilizing *SPSS 26*, *Amos 22* and *GraphPad Prism 9*.

**FIGURE 3:**
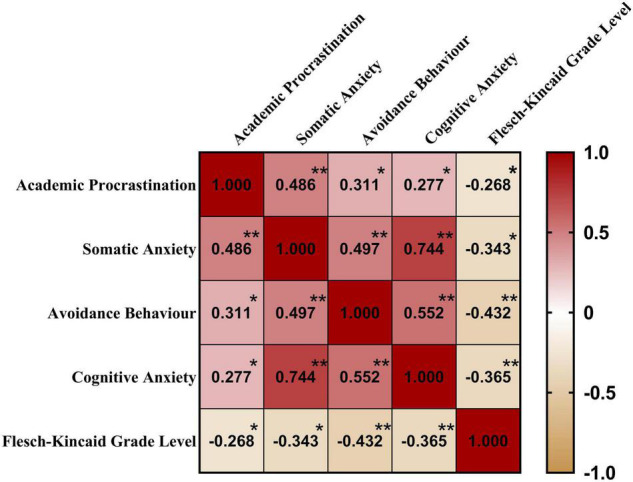
Heat map: correlations among academic procrastination, L2 writing anxiety and Flesch-Kincaid Grade Level. Values in the map are correlation coefficients; * *Sig* < 0.05; ** *Sig* < 0.01.

Figure 3 displays the correlations among the variables in the current study, including academic procrastination, L2 writing anxiety and Flesch-Kincaid Grade Level. This chart is revealing in that academic procrastination was significantly positively correlated with the dimensions of L2 writing anxiety but was significantly negatively correlated with Flesch-Kincaid Grade Level; the dimensions of L2 writing anxiety were significantly negatively correlated with Flesch-Kincaid Grade Level.

Tables 1, 2 present the results of regression analysis which probed into the impact of academic procrastination on L2 writing anxiety, and the impact of L2 writing anxiety on Flesch-Kincaid Grade Level. Results suggested that academic procrastination can significantly influence somatic anxiety, avoidance behavior, and cognitive anxiety in L2 writing in a positive way. Meanwhile, all the three dimensions of L2 writing anxiety can significantly affect Flesch-Kincaid Grade Level negatively.

**TABLE 1:** The impact of academic procrastination on L2 writing anxiety: regression analysis.

		Academic procrastination (Independent variable)	
		*F*	*p* value
L2 writing anxiety (Mediating variable)	Somatic anxiety	16.424	0.000**
	Avoidance behavior	5.683	0.021*
	Cognitive anxiety	4.400	0.041*

*p < 0.05; **p < 0.01.

**TABLE 2:** The impact of L2 writing anxiety on Flesch-Kincaid Grade Level: regression analysis.

	L2 writing anxiety (Mediating variable)					
	Somatic anxiety		Avoidance behavior		Cognitive anxiety	
	*F*	*p* value	*F*	*p* value	*F*	*p* value
Flesch-Kincaid Grade Level (Dependent variable)	7.051	0.010*	12.182	0.001**	8.136	0.006**

*p < 0.05; **p < 0.01.

**FIGURE 4:**
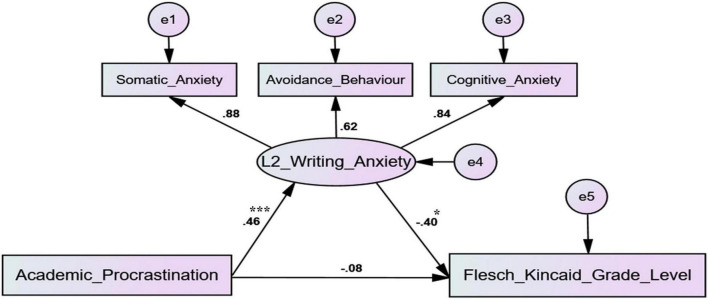
The structural equation model. *p < 0.05; ***p < 0.001.

Based on the correlation and regression analyses, *Amos 22* was adopted to construct the structural equation model (SEM) and perform data fitting, as shown in Figure 4. Table 3 indicates that the majority of indices of SEM were within the reference range, which provided solid evidence for the acceptability of the constructed model.

**TABLE 3:** Data fitting in the structural equation model.

Indicators	CMIN/DF	*p*	GFI	CFI	IFI	RMR
Value	2.508	0.04	0.934	0.929	0.934	0.039
Reference range	≤ 5	> 0.05	≥ 0.9	≥0.9	≥ 0.9	≤ 0.10

As is shown in Figure 4, the path coefficient from academic procrastination to L2 writing anxiety was significant. In the meantime, the path coefficient from L2 writing anxiety and Flesch-Kincaid Grade Level was also significant. However, the path coefficient from academic procrastination to Flesch-Kincaid Grade Level was not significant. Path analysis (see Table 4) revealed that the standardized total effect of academic procrastination on Flesch-Kincaid Grade Level was significant (i.e., 0 was not involved in the 95% confidence interval), and the standardized indirect effect was significant. However, it was striking that the standardized direct effect was not significant (i.e., 0 was involved in the 95% confidence interval). According to the analyzing model of mediating effect proposed by Wen and Ye (2014), L2 writing anxiety played a complete mediating role in the impact of academic procrastination on Flesch-Kincaid Grade Level.

**TABLE 4:** Effect analysis of the paths.

Path	Standardized total effect		Standardized direct effect		Standardized indirect effect	
	95% CI		95% CI		95% CI	
	Lower limit	Upper limit	Lower limit	Upper limit	Lower limit	Upper limit
Academic procrastination→ L2 writing anxiety	0.207	0.688	0.207	0.688	—	—
L2 writing anxiety→ Flesch-Kincaid Grade Level	−0.732	−0.056	−0.732	−0.056	—	—
Academic procrastination→ Flesch-Kincaid Grade Level	−0.450	−0.051	−0.383	0.229	−0.426	−0.019

## Discussion

### The Impact of Academic Procrastination on L2 Writing Performance

This research innovatively introduced academic procrastination, which is a domain-general psychological property, into the context of second language acquisition, and explored the impact of academic procrastination on L2 writing. The results revealed that academic procrastination can negatively influence Flesch-Kincaid Grade Level, which represents the traditional features of text readability in L2 writing, on a significant level. The higher the level of academic procrastination, the lower the average number of syllables and sentence length in L2 writing texts, which indicated a lower level of text difficulty of L2 writing production.

The findings in the present study accorded with earlier observations in the negative impact of academic procrastination on foreign language learning (Zhong, 2017; Aydoğan and Akbarov, 2018; Yurtseven and Akpur, 2018; Qi, 2020) and further verified the findings in Kafipour and Jafari (2021). This research also enriched the methods of L2 writing evaluation and in the meantime elucidated the relationship between academic procrastination and the average number of syllables and sentence length in L2 writing. A smaller number of syllables per word suggested the usages of shorter words. Sentences, whose average sentence length was limited, might have fewer complicated structures such as clauses, etc., and had an insufficient capacity to deliver all-round information. In the teaching context of L2 writing, the passages that L2 learners have written are to be submitted for teachers’ evaluation or peer review and a strict deadline is usually imposed. On account of the fear of negative feedback and peer pressure, or individual perfectionism, L2 learners might irrationally withdraw from writing in a second language. However, as the deadline is approaching, completing the L2 writing task is of the utmost urgency. The learners had no way but to finish writing the essay in their L2 as fast as they can before the deadline expired. Dictions and authentic expression were then not within their concern. Learners can hardly convey themselves clearly since their texts were fragmented by using considerable short vocabularies and sentences, which in turn provide little space for learners to apply their repertoire in L2 writing. Their L2 writing proficiency, thus, can hardly be promoted.

### The Complete Mediating Role of L2 Writing Anxiety

In addition to the exploration of the overall impact of academic procrastination on L2 writing performance, this research also attempted to expound on the mediating role of L2 writing anxiety in the impact. It has been demonstrated that academic procrastination can positively influence all the three dimensions of L2 writing anxiety on a significant level. Guided by the temporal motivation theory (Steel et al., 2018) and the control-value theory (Pekrun, 2006), academic procrastination and anxiety are interdependent, and can both be triggered by expectancy and value appraisals. When a lower level of expectancy is perceived, the value of the task is underrated accordingly. These appraisals would lead to the instigation of anxiety and academic procrastination. L2 learners might experience considerable apprehension in L2 writing tasks, due to their severe academic procrastination. A series of somatic discomfort and unpleasant experiences associated with somatic anxiety was then aroused. As the deadline approaches, cognitive anxiety was aggravated. L2 learners often appraised the excellence of completing the L2 writing task. When they feel that it was less likely to fully apply their repertoire in the writing task, negative expectations were heightened, and they would concern more regarding potential teachers’ evaluation and peer review. Avoidance behavior was thus formed. Results from the current study further support and proffered empirical evidence to the “AAA” theory (Milgram and Toubiana, 1999).

Meanwhile, L2 writing anxiety had a significant negative impact on Flesch-Kincaid Grade Level as the indicator of L2 writing readability. The results further confirmed the negative influence of L2 writing anxiety on L2 writing performances in previous studies (Guo and Qin, 2010; Asmari, 2013; Li et al., 2013; Sarkhoush, 2013; Guo, 2018; Sabti et al., 2019; Saedpanah and Mahmoodi, 2020; Soleimani et al., 2020). Furthermore, this study contributed to enriching the system of L2 writing evaluation and discussed the relationship between L2 writing anxiety and the inherent characteristics of L2 writing texts. According to the Broaden-and-Build theory (Fredrickson, 2001), anxiety could impede the construction of cognitive resources. Given that the total amount of cognitive resources of learners is limited (Skehan, 1998), once anxiety is aroused, their cognitive resources would be constricted. The cognitive load would increase, and the difficulty of language production would decline accordingly.

The structural equation modeling analysis revealed that academic procrastination can indirectly and significantly influence L2 writing performance via L2 writing anxiety. The influential path from academic procrastination to L2 writing was delineated by encompassing L2 writing anxiety as the mediating variable. Severe academic procrastination suggests great anxiety in L2 writing from all three dimensions and indicates a lower level of text difficulty in L2 writing correspondingly.

### Contributions and Pedagogical Implications

The current interdisciplinary study made an initial attempt in exploring the roles of psychological properties in the L2 writing context and was the first study that integrated academic procrastination, anxiety, and text readability as the measure for L2 writing performance. The findings enhanced our understanding of the negative impact that academic procrastination can exert on second language writing. Meanwhile, the present research extended our knowledge of anxiety and shed light on its mediating role in the aforementioned impact. This study also provided strong empirical evidence and represented a major breakthrough in understanding the influential path among the three main variables. Methodologically, the adoption of the structural equation modeling approach contributed to in-depth analysis, thus was a key strength of the present study.

Meanwhile, the results from the current study also provide illuminating insights with regard to pedagogical practices. Firstly, while cultivating foreign language skills, teachers and educational practitioners should pay attention to the psychological state of L2 learners, especially in terms of academic procrastination and anxiety which could significantly influence the L2 performances. It is also necessary to offer individual guidance for learners so that their repertoire in L2 and language learning psychology can be promoted simultaneously. Secondly, the evaluation system of L2 writing needs to be further advanced. Changes can be implemented in the methods of assessment to minimize the negative expectations of writing in L2 from learners. Academic procrastination and anxiety can be kept at a lower level; hence, L2 learners’ performances could be enhanced.

### Limitations and Future Directions

Although the current study has successfully demonstrated the impact of academic procrastination on L2 writing and the mediating effect of L2 writing anxiety within the impact, several limitations to this research need to be acknowledged. Firstly, the sample size was relatively small, due to the restriction of the number of students who shared the same training experience in English writing in the previous stage. The generalizability might be limited. The findings, thus, need to be interpreted with caution. Secondly, albeit a step in the right direction, the present research was based on a cross-sectional design which could only provide a snapshot of the roles of academic procrastination and anxiety in L2 writing. Longitudinal explorations with delayed collections of data are necessary to confirm the impact and generalize over time. Notwithstanding these limitations, this study offers insight into the power of academic procrastination in L2 writing and its influential path and adds to our understanding of how psychological factors perform as the driving force in L2 acquisition and learning. Future works could usefully explore the impact of academic procrastination on other aspects of L2 proficiencies, for instance, speaking, listening and reading in the L2 context. It would also be interesting to assess the effects of other domain-general psychological factors and emotions in L2 acquisition and learning. Another possible area of future research would be to examine the text readability thoroughly by using machine learning models (Lee et al., 2021). The current study particularly adopted Flesch-Kincaid Grade Level in the correlation, regression and SEM analysis. By conducting machine learning analysis in future works, more comprehensive text features can be presented. Furthermore, a mixed method of empirical design is recommended to address the issue from both quantitative and qualitative perspectives, so that more solid evidence can be utilized to contribute to the analysis.

## Conclusion

The current research broke through the barriers between psychology and L2 writing, by introducing the psychological properties of academic procrastination and anxiety to the study. L2 writing readability was innovatively utilized to assess the text difficulty in L2 writing. By exploring the relationship between academic procrastination and L2 writing performance, this study demonstrated the significant negative impact of academic procrastination on the Flesch-Kincaid Grade Level of L2 writing and clarified the mediating effect of L2 writing anxiety in the impact. The influential path was also established and delineated. Overall, the lower levels of academic procrastination and anxiety, which suggest positive psychology in L2 writing, could indicate a higher level of L2 English writing production.

## Data Availability Statement

The original contributions presented in the study are included in the article/supplementary material, further inquiries can be directed to the corresponding author.

## Ethics Statement

The studies involving human participants were reviewed and approved by College of Foreign Languages, Nankai University. The patients/participants provided their written informed consent to participate in this study.

## Author Contributions

CZ contributed to the conceptualization of the study, literature review, data collection and analysis, and writing the first draft. WZ contributed to the conceptualization of the study and reviewing and editing. Both authors contributed to manuscript revision, read, and approved the submitted version.

## Conflict of Interest

The authors declare that the research was conducted in the absence of any commercial or financial relationships that could be construed as a potential conflict of interest.

## Publisher’s Note

All claims expressed in this article are solely those of the authors and do not necessarily represent those of their affiliated organizations, or those of the publisher, the editors and the reviewers. Any product that may be evaluated in this article, or claim that may be made by its manufacturer, is not guaranteed or endorsed by the publisher.
